# Intraoperative hypotension and short-term outcomes after primary total hip arthroplasty: a retrospective cohort study

**DOI:** 10.1302/2633-1462.76.BJO-2026-0009.R1

**Published:** 2026-06-09

**Authors:** Alexander Burbelo, Trace Clark, Andrew Blake Huffman, Nicholas Scarl, Stephanie O’Palko, Brian Ferguson, Jay Shepherd, Matthew Bullock

**Affiliations:** 1 Department of Orthopaedic Surgery, Marshall University, Joan C. Edwards School of Medicine, Huntington, West Virginia, USA; 2 Department of Anesthesiology, Marshall University, Joan C. Edwards School of Medicine, Huntington, West Virginia, USA

**Keywords:** Total hip arthroplasty, Intraoperative hypotension, Anaesthesia, Length of stay, Hospital readmission, primary total hip arthroplasty, intraoperative hypotension, hypotension, revision surgery, blood loss, postoperative complications, total hip arthroplasties (THAs), intraoperative blood loss, Multivariable logistic regression, anaesthesiologists

## Abstract

**Aims:**

Intraoperative hypotension (IOH) has been associated with adverse outcomes in non-cardiac surgery, but its effect during total hip arthroplasty (THA) remains unclear. This study aimed to investigate whether absolute (AIOH) or relative (RIOH) IOH is a risk factor for adverse short-term outcomes following primary THA.

**Methods:**

A retrospective review of 394 THAs (394 patients) performed at a single academic institution between September 2020 and April 2025 was conducted. AIOH was defined as any intraoperative mean arterial pressure (MAP) < 65 mmHg, and RIOH as a ≥ 20% decrease from preoperative baseline MAP. Primary outcomes included all-cause 30- and 90-day readmissions and postoperative length of stay (LOS). Multivariable logistic regression was used to identify predictors of readmission and LOS > one day.

**Results:**

Overall, 112 patients (28.4%) experienced AIOH during their THA. Neither the presence nor duration of AIOH was associated with readmission or LOS. RIOH occurred in 74 patients (22.5%), and was not associated with readmission or LOS, but was associated with higher rates of reoperation within 90 days (12.2% vs 2.4%, p = 0.001). Estimated blood loss independently predicted prolonged LOS across all models (odds ratio 1.75 per 50 ml, 95% CI 1.33 to 2.31, p < 0.001).

**Conclusion:**

AIOH was not associated with an increased risk of adverse short-term outcomes following primary THA when defined by a fixed MAP threshold. RIOH and intraoperative blood loss demonstrated stronger associations with postoperative complications, but these findings require further evaluation. This study supports a more individualized approach to haemodynamic monitoring in primary THA.

Cite this article: *Bone Jt Open* 2026;7(6):766–776.

## Introduction

An estimated 544,000 total hip arthroplasties (THAs) are performed annually in the USA.^1^ This number is projected to increase substantially, with primary THA procedures expected to rise by 174% to approximately 572,000 surgeries annually by 2030.^2^ THA provides notable improvements in mobility and quality of life; however, as procedural volume continues to grow, optimizing perioperative care to minimize complications becomes increasingly critical.

Complications following elective hip arthroplasty are relatively uncommon. One study examining nearly 100,000 THAs reported an overall complication rate of 4.9%.^3^ However, another study found that 13.4% of patients had at least one Emergency Department (ED) visit following THA, most commonly related to pain or swelling.^4^ Similarly, common reasons for failure to achieve same-day discharge after outpatient THA include nausea, vomiting, and postoperative urinary retention.^5^

Numerous perioperative and patient-related risk factors have also been associated with length of stay.^6^ Overall, infection remains the most common cause of surgical readmission.^4^ Hospitals with higher 30-day readmission rates following THA are more likely to experience greater revenue losses,^7^ underscoring the financial incentive to optimize perioperative care protocols. This includes recent investigations evalulating the necessity of routine postoperative laboratory testing after THA.^8^

While traditional risk factors for complications following hip arthroplasty, such as obesity, diabetes, and smoking, are well recognized, intraoperative haemodynamic and physiological factors have been less thoroughly studied.^9-11^ Intraoperative hypotension (IOH) has emerged as a potentially modifiable contributor to adverse outcomes.^12^

Although more than 100 definitions of IOH exist, it is most commonly characterized as either absolute IOH (AIOH), defined as a mean arterial pressure (MAP) < 65 mmHg, or relative IOH (RIOH), defined as ≥ 20% decrease from baseline MAP.^13^ IOH is often employed intentionally to reduce intraoperative blood loss during THA.^14,15^ However, prolonged IOH has been associated with end-organ hypoperfusion and an increased risk of postoperative complications, including acute kidney injury, myocardial infarction, and mortality.^16,17^ In orthopaedic and thoracic surgery, IOH lasting longer than five minutes has been linked to an increased risk of postoperative delirium in elderly patients.^18^

Additionally, in elective shoulder arthroplasty, patients receiving general anaesthesia were significantly more likely to experience IOH and demonstrated poorer ambulatory outcomes, including reduced rates of same-day discharge.^19^ However, the relationship between IOH and postoperative complications in the context of THA remains underinvestigated.

The premise that IOH impairs tissue perfusion and immune function, thereby increasing the risk of postoperative complications, is supported by both observational and mechanistic evidence.^20,21^ Consequently, hypotension during THA may increase the risk of postoperative readmission by compromising host defence mechanisms and impairing healing capacity.

The purpose of this study is to evaluate whether AIOH or RIOH is associated with an increased incidence of 90-day readmission following primary THA, as well as prolonged length of stay (LOS).

## Methods

### Patient cohort

A retrospective review was conducted of 501 adult patients who underwent THA performed by three fellowship-trained arthroplasty surgeons and multiple board-certified anaesthesiologists at a single academic tertiary care institution between September 2020 and April 2025. Patients aged 18 years or older were included if they had complete intraoperative records and were followed for a minimum of 90 days postoperatively. Patients were excluded if they underwent revision surgery (n = 75), had a non-THA index procedure (n = 17), underwent surgery for a nonelective indication such as a hip fracture (n = 10), had a non-THA trauma procedure (n = 4), or underwent surgery as part of a polytrauma case (n = 1). After applying these exclusion criteria, a total of 394 patients were included in the final analysis. The study included patients regardless of surgical approach, with 93.9% of the final cohort undergoing an anterior approach. Cemented THAs comprised a small minority of cases and were therefore retained in analyses to preserve statistical power. Institutional review board approval was obtained prior to study initiation. Given the retrospective nature of the study and the use of de-identified data, the requirement for informed consent was waived. This study was conducted and reported in accordance with the STROBE guidelines for observational studies.^22^

### Patient characteristics

Among the 394 patients included in this study, 112 (28.4%) experienced AIOH, while the remaining 282 (71.6%) did not meet the criteria (Table I).

**Table I.** Demographic and clinical characteristics by absolute intraoperative hypotension (AIOH).

Variable	Overall (n = 394)	No AIOH (n = 282)	AIOH (n = 112)	p-value
Median age, yrs (IQR)	65.0 (58.0 to 72.0)	64.0 (57.2 to 71.0)	67.0 (59.0 to 73.0)	0.173*
Median BMI, kg/m² (IQR)	31.65 (28.05 to 36.85)	31.8 (27.9 to 36.8)	31.3 (28.4 to 37.4)	0.794*
Sex – male, n (%)	187 (47.5)	134 (47.5)	53 (47.3)	> 0.999†
**ASA grade, n (%)**				0.756†
I	1 (0.3)	1 (0.4)	0 (0.0)	
II	91 (23.1)	68 (24.1)	23 (20.5)	
III	287 (72.8)	203 (72.0)	84 (75.0)	
IV	15 (3.8)	10 (3.5)	5 (4.5)	
Regional anaesthesia, n (%)	301 (76.4)	192 (68.1)	109 (97.3)	< 0.001†
Hypertension, n (%)	292 (74.1)	211 (74.8)	81 (72.3)	0.612†
Coronary artery disease, n (%)	206 (52.3)	147 (52.1)	59 (52.7)	0.580†
Chronic obstructive pulmonary disease, n (%)	66 (16.8)	59 (20.9)	7 (6.2)	0.001†
Diabetes mellitus type 2, n (%)	99 (25.1)	66 (23.4)	33 (29.5)	0.246†
Chronic kidney disease, n (%)	72 (18.3)	56 (19.9)	17 (15.0)	0.316†
Cardiac arrhythmia, n (%)	43 (10.9)	26 (9.2)	17 (15.2)	0.106†
All-cause 90-day readmission, n (%)	26 (6.6)	22 (7.8)	4 (3.6)	0.176†
**Implant fixation, n (%)**				> 0.999†
Cemented	8 (2.0)	6 (2.1)	2 (1.8)	
Uncemented	386 (98.0)	276 (97.9)	110 (98.2)	
**Surgical approach, n (%)**				> 0.999†
Anterior	370 (93.9)	265 (94.0)	105 (93.8)	
Posterior	24 (6.1)	17 (6.0)	7 (6.2)	
Median TXA dose, mg (IV only) (IQR)	1,000 (870 to 1,000)	1,000 (850 to 1,000)	1,000 (900 to 1,000)	0.325*
**TXA route, n (%)**				0.876†
IV	352 (89.3)	251 (89.0)	101 (90.2)	
Topical	42 (10.7)	29 (10.3)	13 (11.6)	

AIOH = any intraoperative mean arterial pressure < 65 mmHg.

* Mann-Whitney U test.

† Fisher’s exact test.

ASA, American Society of Anesthesiologists; IV, intravenous; TXA, tranexamic acid.

### Surgical and anaesthetic technique

Blood conservation during primary THA at our institution typically includes routine intravenous tranexamic acid (TXA) administration and contemporary haemostatic techniques without the use of tourniquets or cell salvage. There is no institutional protocol endorsing deliberate hypotensive anaesthesia. Intraoperative MAP is maintained within standard anaesthetic parameters at the discretion of the attending anaesthesiologist.

Spinal anaesthesia is used unless contraindicated or unsuccessful, in which case patients receive general endotracheal anaesthesia (GETA). There is no mandated intrathecal agent or standardized dosing protocol across patients. Antihypertensive management remained consistent throughout the study period: beta-blockers and calcium-channel blockers were typically continued, whereas angiotensin-converting enzyme inhibitors and angiotensin receptor blockers were withheld on the day of surgery.

There is no standardized institutional protocol for intraopartive fluid resuscitation specific to primary THA; fluid management (crystalloid and/or colloid) is determined by the attending anaesthesiologist. Similarly, there is no formal preoperative hydration protocol or standardized fluid-loading strategy. Case timing during the day was not standardized with regard to perioperative fluid management.

### Exposure technique

Baseline MAP was defined as the first MAP recorded in the intraoperative anaesthesia record at case initiation (prior to anaesthetic administration). MAP was subsequently monitored continuously at automated one-minute intervals throughout the procedure.

Patients who experienced a MAP < 65 mmHg at any point during surgery were classified as having AIOH, and the cumulative duration of hypotension was recorded. In addition to the binary definition, the cumulative duration of hypotension was evaluated in secondary analyses. RIOH was also assessed and defined as ≥ 20% decrease in the intraoperative geometrical mean MAP at any timepoint compared with baseline preoperative MAP. The RIOH analysis was limited to patients with complete baseline MAP data (n = 329).

### Data collection

Patient demographic data and baseline characteristics, including age, sex, BMI, American Society of Anesthesiologists (ASA) grade,^23^ and anaesthesia type (general vs spinal) were extracted from the electronic health record. Elixhauser comorbidities were derived for each patient using International Classification of Diseases of the World Health Organization, tenth revision (ICD-10) codes.^24^ Perioperative surgical variables, including intraoperative MAP and procedure duration, were obtained from the anaesthesia record for each case. Estimated blood loss was determined by visual assessment and intraoperative estimation performed by the attending surgeon for each case. Primary outcome measures included all-cause 30- and 90-day readmissions and LOS, which were adjudicated through chart review of clinician documentation and categorized accordingly.

### Statistical analysis

Continuous variables were assessed for normality using the Shapiro-Wilk test. Non-normally distributed variables were reported as medians with IQRs and compared using the Mann-Whitney U test. Categorical variables were summarized as counts with percentages and compared using Fisher’s exact test. Patients were stratified by the presence or absence of AIOH and RIOH for univariate comparisons. Univariate analyses of demographic and perioperative characteristics were also performed to compare patients with and without readmission.

Multivariable logistic regression was used to evaluate independent predictors of postoperative LOS > one day, with separate models constructed for AIOH and RIOH. Secondary analyses treated AIOH and RIOH as duration-based variables in ten-minute increments. Non-linear associations between hypotension duration and LOS were assessed using orthogonal polynomial contrasts (linear, quadratic, and cubic). Sensitivity analyses were conducted using Poisson regression to model LOS as a count outcome. Regression results are reported as adjusted odds ratios (ORs) or incidence rate ratios (IRRs) with corresponding 95% CIs. All statistical analyses were performed using R v. 4.4.3 (RStudio; Posit, USA). A value of p < 0.05 was considered statistically significant.

## Results

Patients experiencing AIOH were significantly more likely to have received regional anaesthesia (97.3% vs 68.1%, p < 0.001, Fisher’s exact test; Table I). There were no significant differences between groups for most comorbidities, however the prevalence of chronic obstructive pulmonary disease (COPD) was significantly higher in the non-IOH group (20.9% vs 6.2%, p = 0.001, Fisher’s exact test). Given the number of baseline comparisons performed and the absence of adjustment for multiple testing, this observed difference in COPD prevalence may represent a chance finding rather than a clinically meaningful imbalance and should be interpreted cautiously. No significant differences were observed between groups in ASA grade, implant fixation method, or surgical approach.

Baseline MAP, operating time, and estimated blood loss were similar between patients with or without AIOH (all p > 0.500) (Table II). Patients in the AIOH group were significantly more likely to require intraoperative vasopressor support compared with those without AIOH (90.2% vs 53.2%, p < 0.001, Fisher’s exact test). No significant differences were observed between groups for perioperative red blood cell transfusions, 30-day readmissions, 90-day readmissions, or reoperation within 90 days (all p > 0.170). Median length of stay was 1.0 day in both groups, with similar LOS distributions. In multivariable analysis, AIOH was not associated with an increased likelihood of LOS greater than 1.0 day (OR 1.35, 95% CI 0.61 to 2.87, p = 0.450, Fisher’s exact test).

**Table II.** Perioperative characteristics and outcomes by absolute intraoperative hypotension.

Variable	No AIOH (n = 282)	AIOH (n = 112)	p-value
Median baseline MAP, mmHg (IQR)	103.0 (90.0 to 118.2)	104.2 (92.4 to 111.9)	0.537*
Median operating time, hrs (IQR)	1.5 (1.4 to 1.9)	1.6 (1.4 to 1.8)	0.545*
Median estimated blood loss, cc (IQR)	300 (250 to 300)	300 (250 to 300)	0.762*
Any vasopressor use, n (% yes)	150 (53.2)	101 (90.2)	< 0.001†
Perioperative RBC transfusion, n (% yes)	3 (1.1)	0 (0.0)	0.561†
Median LOS, days (IQR)	1.0 (1.0 to 1.0)	1.0 (1.0 to 1.0)	0.405*
LOS > 1 day, n (%)	25 (8.9)	13 (11.6)	0.450†
30-day readmission, n (%)	16 (5.7)	3 (2.7)	0.299†
Any 90-day readmission, n (%)	22 (7.8)	4 (3.6)	0.176†
Reoperation within 90 days, n (%)	13 (4.6)	2 (1.8)	0.27†

AIOH = any intraoperative MAP < 65 mmHg.

* Mann-Whitney U test.

† Fisher’s exact test.

AIOH, absolute intraoperative hypotension; LOS, length of stay; MAP, mean arterial pressure; RBC, red blood cell.

Of the 394 patients, 26 (6.6%) experienced a 90-day readmission. Demographic characteristics, including sex, age, and BMI, were similar between patients with and without readmission (Table III). Although the median LOS (1.0 day) and estimated blood loss (EBL, 300 ml) were comparable between groups, the overall distributions of LOS (p = 0.011, Mann-Whitney U test) and EBL (p = 0.002, Mann-Whitney U test) differed significantly (Table III). Intensive care unit-level care was required for five patients (19.2%), of whom only one met the criteria for both absolute and relative IOH during their THA (Supplementary Material).

**Table III.** Demographic and clinical characteristics by 90-day readmission.

Variable	No readmission (n = 368)	Any 90-day readmission (n = 26)	p-value
Sex – female, n (%)	190 (51.6)	17 (65.4)	0.223*
Sex – male, n (%)	178 (48.4)	9 (34.6)	0.223
Anaesthesia – general, n (%)	88 (23.9)	5 (19.2)	0.811*
Anaesthesia – regional, n (%)	280 (76.1)	21 (80.8)	0.811
**ASA grade, n (%)**			0.117
I	1 (0.3)	0 (0.0)	
II	88 (23.9)	3 (11.5)	
III	267 (72.6)	20 (76.9)	
IV	12 (3.3)	3 (11.5)	
Hypertension, n (%)	270 (73.4)	22 (84.6)	0.252*
Coronary artery disease, n (%)	195 (53.0)	11 (42.3)	0.316*
COPD, n (%)	65 (17.7)	1 (3.8)	0.098*
Diabetes mellitus type 2, n (%)	154 (41.8)	13 (50.0)	0.420*
Chronic kidney disease, n (%)	68 (18.5)	5 (19.2)	> 0.999*
Cardiac arrhythmia, n (%)	46 (12.5)	2 (7.7)	0.756*
Any vasopressor use, n (% yes)	232 (63.0)	19 (73.1)	0.400*
Perioperative RBC transfusion, n (% yes)	2 (0.5)	1 (3.8)	0.186*
Median age, yrs (IQR)	65.0 (58.0 to 71.0)	70.5 (60.5 to 75.8)	0.142†
Median BMI, kg/m² (IQR)	31.6 (27.8 to 36.8)	32.8 (29.5 to 37.7)	0.416†
Median baseline MAP, mmHg (IQR)	102.7 (90.7 to 115.5)	105.8 (92.5 to 111.9)	0.981†
Median operating time, hrs (IQR)	1.6 (1.4 to 1.8)	1.6 (1.4 to 2.0)	0.286†
Median length of stay, days (IQR)	1.0 (1.0 to 1.0)	1.0 (1.0 to 1.0)	0.011†
Median estimated blood loss, cc (IQR)	300 (250 to 300)	300 (299 to 350)	0.002†

* Fisher’s exact test.

† Mann-Whitney U test.

ASA, American Society of Anesthesiologists; COPD, chronic obstructive pulmonary disease; MAP, mean arterial pressure; RBC, red blood cell.

Among the 26 patients readmitted within 90 days, 15 (3.8% of the original cohort) required reoperation. Indications for reoperation included periprosthetic joint infection (n = 6), dislocation (n = 3), periprosthetic or traumatic fracture (n = 5), and systemic causes (n = 1). Of the 15 patients that underwent reoperation, ten (66.7%) had experienced RIOH during their primary THA, whereas only two patients (13.3%) had experienced AIOH (Supplementary Material).

Assessment of RIOH (≥ 20% decrease from baseline MAP) identified 74 patients (22.5%) (Table IV). Compared with patients without RIOH, those with RIOH had significantly higher prevalence of coronary artery disease (66.2% vs 49.8%, p = 0.017), COPD (31.1% vs 12.5%, p < 0.001), and chronic kidney disease (29.7% vs 14.9%, p = 0.006), and were more likely to receive general anaesthesia (54.1% vs 16.5%, p < 0.001). In contrast, type 2 diabetes mellitus was less common among patients with RIOH (31.1% vs 44.3%, p = 0.045). Patients experiencing RIOH were younger (p = 0.037), had lower baseline MAP (p < 0.001), and longer operating times (p < 0.001). No significant differences were observed in intraoperative vasopressor use, perioperative red blood cell transfusions, hospital LOS, EBL, or readmission rates (all p > 0.05). However, reoperation within 90 days was significantly more frequent in the RIOH group (12.2% vs 2.4%, p = 0.001).

**Table IV.** Characteristics by relative hypotension.

Variable	RIOH present (n = 74)	No RIOH (n = 255)	p-value
Sex – female, n (%)	41 (55.4)	139 (54.5)	> 0.999*
Sex – male, n (%)	33 (44.6)	116 (45.5)	> 0.999*
Anaesthesia – general, n (%)	40 (54.1)	42 (16.5)	< 0.001*
Anaesthesia – regional, n (%)	34 (45.9)	213 (83.5)	< 0.001*
**ASA grade, n (%)**			0.006*
I	1 (1.4)	0 (0.0)	
II	25 (33.8)	52 (20.4)	
III	48 (64.9)	192 (75.3)	
IV	0 (0.0)	11 (4.3)	
Hypertension, n (%)	52 (70.3)	193 (75.7)	0.365*
Coronary artery disease, n (%)	49 (66.2)	127 (49.8)	0.017*
COPD, n (%)	23 (31.1)	32 (12.5)	< 0.001*
Chronic kidney disease, n (%)	22 (29.7)	38 (14.9)	0.006*
Diabetes mellitus type 2, n (%)	23 (31.1)	113 (44.3)	0.045*
Cardiac arrhythmia, n (%)	7 (9.5)	34 (13.3)	0.430*
Any vasopressor use, n (% yes)	40 (54.1)	169 (66.3)	0.074*
Perioperative RBC transfusion, n (% yes)	1 (1.4)	2 (0.8)	0.536*
30-day readmission, n (% yes)	5 (6.8)	12 (4.7)	0.550*
90-day readmission, n (% yes)	2 (2.7)	6 (2.4)	> 0.999*
Reoperation within 90 days, n (% yes)	9 (12.2)	6 (2.4)	0.001*
Median age, yrs (IQR)	63.0 (55.0 to 70.0)	66.0 (59.0 to 73.0)	0.037†
Median BMI, kg/m² (IQR)	32.1 (28.4 to 36.8)	31.3 (27.6 to 35.7)	0.406†
Median baseline MAP (preop), mmHg (IQR)	84.8 (78.5 to 89.6)	108.0 (98.8 to 119.3)	< 0.001†
Median operating time, hrs (IQR)	1.8 (1.5 to 2.1)	1.6 (1.4 to 1.8)	< 0.001†
Median length of stay, days (IQR)	1.0 (1.0 to 1.0)	1.0 (1.0 to 1.0)	0.758†
Median estimated blood loss, cc (IQR)	300 (250 to 300)	300 (250 to 300)	0.567†

RIOH = intraoperative geometric-mean MAP (GEOMEAN) < 80% of preoperative MAP.

* Fisher’s exact test.

† Mann-Whitney U test.

ASA, American Society of Anesthesiologists; COPD, chronic obstructive pulmonary disease; MAP, mean arterial pressure; RBC, red blood cell; RIOH, relative intraoperative hypotension.

In multivariable analyses, EBL was independently associated with increased odds of prolonged LOS in both models. In the AIOH model, EBL was associated with higher odds of LOS > one day (OR 1.75 per 50 ml, 95% CI 1.33 to 2.31, p < 0.001), with no other significant predictors. In the RIOH model, EBL remained an independent predictor (OR 1.76 per 50 ml, 95% CI 1.26 to 2.52, p = 0.001), while male sex was associated with lower odds of prolonged LOS (OR 0.33, 95% CI 0.11 to 0.84, p = 0.027) (Table V). Neither AIOH or RIOH was independently associated with LOS > 1.0 day (p = 0.773 and p = 0.765, respectively). However, ASA grade was independently associated with LOS > 1.0 day in both the AIOH (p = 0.007, Table VI) and RIOH (p = 0.024, Table V) models.

**Table V.** Multivariable predictors of length of stay > one day (relative intraoperative hypotension: > 20% mean arterial pressure decrease).

Variable	Level	OR	95% CI	p-value
Relative hypotension category (> 20% drop)	—	—	—	0.765
ASA grade	—	—	—	0.0243
Age (per year)	—	1.00	0.96 to 1.05	0.873
BMI, kg/m²	—	0.98	0.91 to 1.04	0.568
Sex	Male vs female	0.33	0.11 to 0.84	0.027
Anaesthesia type	Regional vs general	0.80	0.26 to 2.62	0.699
Hypertension	Yes vs no	1.06	0.35 to 3.59	0.924
Coronary artery disease	Yes vs no	1.09	0.43 to 2.76	0.850
Chronic obstructive pulmonary disease	Yes vs no	0.76	0.15 to 2.87	0.713
Chronic kidney disease	Yes vs no	0.46	0.10 to 1.67	0.274
Diabetes mellitus type 2	Yes vs no	1.22	0.48 to 3.06	0.677
Cardiac arrhythmia	Yes vs no	1.20	0.30 to 4.08	0.783
Any vasopressor use	Yes vs no	1.86	0.69 to 5.63	0.243
Estimated blood loss (per 50 ml)	—	1.76	1.26 to 2.52	0.001
Operating time, hours	—	0.58	0.23 to 1.38	0.235

Values are adjusted ORs with 95% CIs. Relative hypotension category and ASA grade evaluated as multilevel categorical variables (p-value = global test). Outcome: length of stay > one day.

ASA, American Society of Anesthesiologists; OR, odds ratio.

**Table VI.** Multivariable predictors of length of stay > 1 day (absolute intraoperative hypotension: mean arterial pressure < 65 mmHg).

Variable	Level	OR	95% CI	p-value
IOH duration category (MAP < 65 mmHg)	—	—	—	0.773
ASA grade	—	—	—	0.0068
Age (per year)	—	1.01	0.97 to 1.05	0.654
BMI, kg/m²	—	0.96	0.90 to 1.02	0.224
Sex	Male vs female	0.49	0.21 to 1.10	0.093
Anaesthesia type	Regional vs general	0.89	0.32 to 2.70	0.832
Hypertension	Yes vs no	1.12	0.41 to 3.35	0.830
Coronary artery disease	Yes vs no	1.10	0.48 to 2.52	0.815
Chronic obstructive pulmonary disease	Yes vs no	0.76	0.20 to 2.35	0.661
Chronic kidney disease	Yes vs no	0.53	0.14 to 1.63	0.304
Diabetes mellitus type 2	Yes vs no	1.49	0.66 to 3.43	0.339
Cardiac arrhythmia	Yes vs no	1.11	0.32 to 3.40	0.859
Any vasopressor use	Yes vs no	1.89	0.75 to 5.22	0.192
Estimated blood loss (per 50 ml)	—	1.75	1.33 to 2.31	< 0.001
Operating time, hours	—	0.78	0.34 to 1.68	0.533

Values are adjusted ORs with 95% CIs. IOH duration category and ASA grade evaluated as multilevel categorical variables (p-value = global test). Outcome: length of stay > 1 day.

ASA, American Society of Anesthesiologists; IOH, intraoperative hypotension; MAP, mean arterial pressure; OR, odds ratio.

**Fig. 1:**
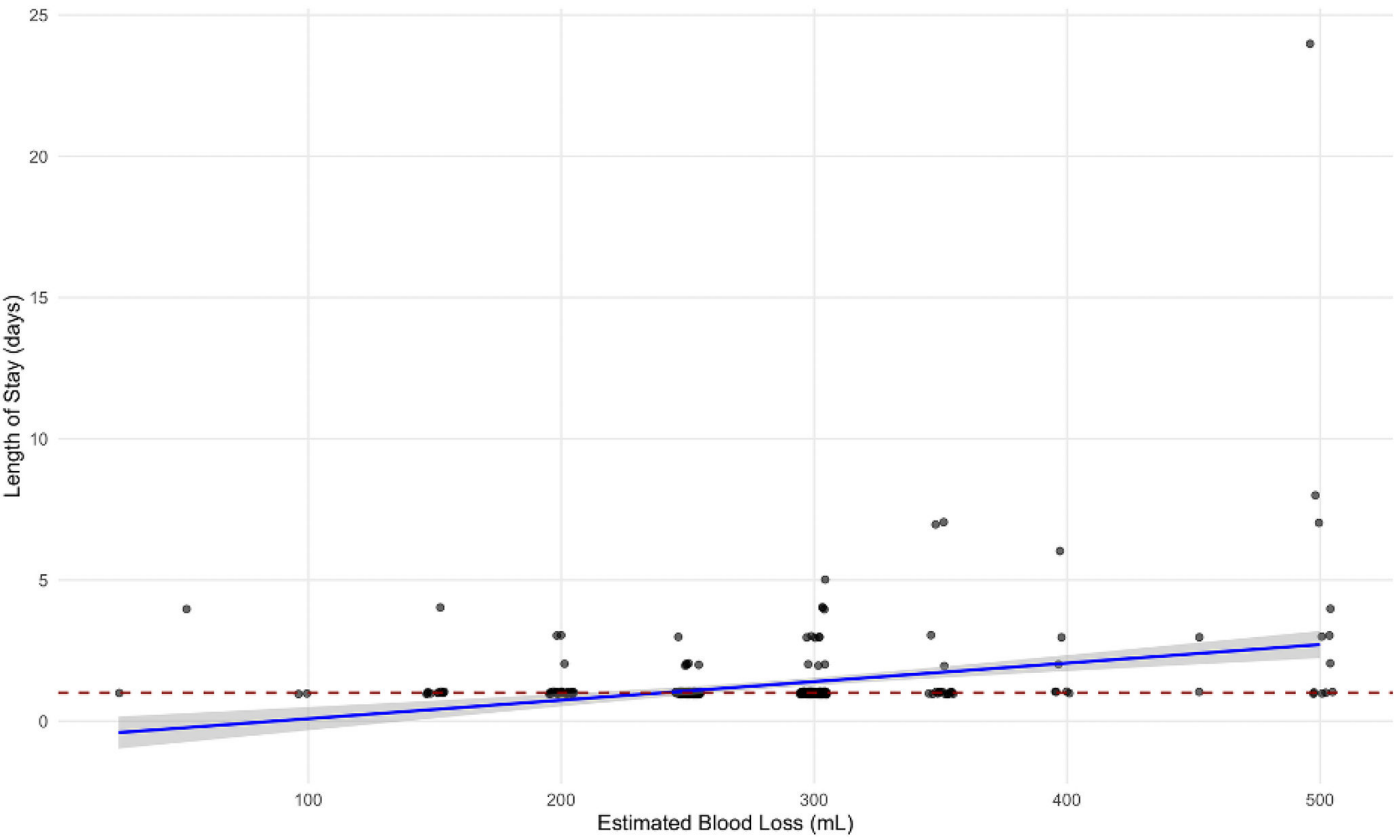
Relationship between estimated blood loss (EBL) and length of stay (LOS). Dots represent individual patients’ EBL on the x-axis and LOS on the y-axis. Solid blue regression line reflects the linear relationship between EBL and LOS with 95% confidence shading. Dashed horizontal line marks the cohort median LOS (1 day).

Secondary regression analyses similarly demonstrated no independent association between the duration of IOH (both AIOH and RIOH) and LOS when evaluated using logistic contrasts or Poisson regression (all p > 0.400). EBL remained a significant predictor of LOS across secondary analyses. The positive association between EBL and postoperative LOS is demonstrated in Figure 1.

## Discussion

In this retrospective cohort study of 394 patients undergoing primary THA, neither AIOH nor RIOH was associated with an increased risk of all-cause 90-day readmissions or prolonged LOS, whether analyzed as a binary or duration-based variable. However, patients who experienced RIOH had a significantly higher rate of reoperation within 90 days, and increased EBL was an independent predictor of prolonged LOS. These findings likely reflect underlying surgical complexity or differences in patient characteristics, rather than independent effects of intraoperative haemodynamics. Given that primary THA typically involves relatively short operating times (usually 60 to 90 minutes), limited physiological stress, and rapid postoperative discharge, transient reductions in MAP may have fewer downstream consequences compared with major revision surgeries. Nevertheless, prior studies have demonstrated that absolute MAP thresholds are linked to end-organ injury and that time-weighted hypotension may serve as a predictor of adverse outcomes in non-cardiac surgical populations.^16^^25^ While most literature on IOH focuses on inpatients undergoing major procedures, THA is increasingly performed in the outpatient setting, raising questions about how intraoperative physiological insults manifest after hospital discharge.

In our cohort, patients who experienced AIOH were significantly more likely to have received regional spinal anaesthesia, whereas patients in the RIOH group were more likely to have undergone general anaesthesia. These associations likely reflect institutional practice patterns and patient selection rather than a causal effect of either anaesthetic technique; accordingly, these findings do not directly imply differences in tissue perfusion. Nevertheless, based on our findings and prior reports, the potential advantage of regional over general anaesthesia in maintaining more consistent perfusion during THA warrants further investigation.^26,27^ Routine use of invasive or continuous blood pressure monitoring may provide additional benefits in elective arthroplasty populations by enabling earlier recognition and more effective management of hypotension.

In multivariable analyses, AIOH was not significantly associated with comorbidities, such as pre-existing hypertension, chronic kidney disease (CKD), diabetes mellitus (DM), coronary artery disease (CAD), or arrhythmia. However, patients who experienced AIOH were significantly more likely to require intraoperative vasopressor administration. Vasopressors are routinely used during surgery to raise blood pressure during hypotensive episodes and maintain adequate organ perfusion. The observed association between AIOH and vasopressor use in this study likely reflects the severity of hypotensive episodes experienced by these patients. While prior studies have examined predictors of vasopressor use in total knee arthroplasty,^28^ there is a paucity of research investigating vasopressor use in total joint replacement as a potentially modifiable factor influencing postoperative outcomes.

RIOH was not associated with increased readmission rates or prolonged LOS, but it was observationally linked to higher rates of reoperation within 90 days. The mechanism underlying this association remains unclear. It is possible that relative MAP reductions reflect underlying physiological vulnerability, impaired vascular tone, or reduced haemodynamic reserve, which may increase susceptibility to postoperative complications even when absolute MAP targets are maintained. These findings support emerging concerns that absolute MAP thresholds alone may not fully capture intraoperative risk and that relative changes from baseline may provide a more individualized assessment of haemodynamic tolerance.^12^ Importantly, baseline MAP values differed between groups, with higher baseline pressures observed in patients who met criteria for RIOH. This difference may represent residual confounding and should be considered when interpreting these findings. One potential solution to stabilize perioperative haemodynamics is the use of preoperative fluid administration, which prior studies have shown to decrease the incidence of haemodynamic instability.^29,30^ While the administration of a presurgical fluid bolus has been shown to successful, there is no standardized methodology or criteria for bolus size or use, as one study adjusted the amount of fluid based on lean mass,^30^ and another used a standardized dose of 500 ml on patients who had a collapsible inferior vena cava.^31^

Although modelling the duration of hypotension for both AIOH and RIOH in the present study demonstrated no consistent association with postoperative outcomes, prior literature suggests that both the magnitude and duration of hypotension may contribute to end-organ dysfunction; however, the available evidence remains limited.^13^ Therefore, future analyses that more precisely characterize time-under-threshold dynamics may better elucidate dose–response relationships between intraoperative hypotension and outcomes following THA.

Increased EBL consistently predicted a prolonged hospital LOS, independent of AIOH or RIOH exposure, across both logistic and Poisson regression models. Although statistically significant differences in LOS and EBL distributions were observed in univariate analyses, statistical significance does not necessarily equate to clinical significance. This association remained significant after adjusting for anaesthesia type, comorbidities, operating time, and vasopressor use, suggesting that blood loss, rather than intraoperative MAP reductions, may be a more prominent contributor to delayed discharge following THA. Prior studies in other surgical populations have similarly linked higher EBL to increased morbidity, reoperation, and mortality,^32,33^ as well as longer LOS,^34^ consistent with the findings of this study. While EBL has been identified as a predictor of postoperative outcomes, its reliability remains debated. No standardized method for quantifying blood loss exists, and the most commonly used technique, visual estimation, is frequently inaccurate.^35-37^

This study has several limitations. As a single-centre, retrospective analysis, the findings may not be generalizable to all THA populations. EBL measurements may be imprecise, introducing potential bias. Although IOH was modelled both as absolute and relative values, hypotension at alternative thresholds could not be evaluated, which may have influenced the results. Our final cohort included 93.9% anterior approach THA patients, however, in theory, when a posterior approach is used in the lateral decubitus position, vena caval compression could represent an additional unaccounted-for variable in this study.

Despite adjustment for vasopressor use and intraoperative blood loss, residual confounding from unmeasured perioperative variables may have influenced the results. Prior studies have demonstrated associations between IOH and specific end-organ complications, including stroke, acute kidney injury, and postoperative delirium; however, these outcomes were not explicitly evaluated in the present analysis. Accordingly, these findings do not exclude the possibility that IOH may affect such outcomes following THA. Future investigations incorporating biomarker-based endpoints or formal cognitive assessments may better characterize these relationships in arthroplasty populations.

The sample size may also have been insufficient to detect clinically meaningful differences in LOS or readmission rates, representing an important limitation. LOS demonstrated a substantial floor effect (median one day (IQR 1 to 1) in both cohorts); therefore, prolonged LOS was defined as > one day. Prolonged LOS occurred in 8.9% of patients without AIOH and 11.6% of those with AIOH. Post-hoc power to detect this observed absolute difference of 2.7% was low (approximately 13%). With the available sample sizes, the study had 80% power to detect an absolute difference of approximately 10% to 11% in prolonged LOS rates; smaller differences may not have been detectable.

These findings suggest AIOH may not represent a clinically significant intraoperative risk factor in lower-acuity procedures such as THA, although further investigation is warranted. Future studies should prospectively evaluate perioperative management of antihypertensive medications, particularly renin–angiotensin system inhibitors,^38^ which were withheld on the day of surgery at our institution, as potential modifiers of hypotension risk. EBL also warrants additional investigation to better define its relationship with postoperative complications following THA, and to establish more reliable methods for intraoperative quantification. Moreover, implementation of standardized fluid administration protocols, which are not currently routine for primary THA at our institution, may help mitigate anaesthetic-induced hypotension and improve perioperative haemodynamic stability.

In conclusion, in this retrospective cohort study, neither AIOH nor RIOH was associated with an increased risk of all-cause 90-day readmissions or prolonged LOS following primary THA. Patients who experienced RIOH had higher rates of reoperation within 90 days, though this association may not reflect causality and should be interpreted as exploratory. EBL, rather than intraoperative MAP reductions, emerged as the most consistent predictor of prolonged hospitalization. These findings suggest that absolute MAP thresholds alone may be insufficient to characterize perioperative risk in THA and support the use of a more individualized approach to intraoperative haemodynamic assessment. Larger, prospective studies are needed to validate these results, as the sample size represents a key limitation of this study.

**Take home message**

- In this cohort of primary total hip arthroplasty patients, intraoperative hypotension defined by an absolute MAP threshold (< 65 mmHg) was not associated with increased 90-day readmissions or prolonged hospital stay.

- However, relative hypotension (≥ 20% reduction from baseline MAP) was associated with higher rates of 90-day reoperation, suggesting that patient-specific blood pressure changes may be more clinically relevant than fixed hypotension thresholds.

- These findings support individualized haemodynamic management strategies and highlight estimated blood loss as an important modifiable predictor of postoperative recovery.

## Data Availability

The datasets generated and analyzed in the current study are not publicly available due to data protection regulations. Access to data is limited to the researchers who have obtained permission for data processing. Further inquiries can be made to the corresponding author.
